# Serum connective tissue growth factor is a highly discriminatory biomarker for the diagnosis of rheumatoid arthritis

**DOI:** 10.1186/s13075-017-1463-1

**Published:** 2017-11-22

**Authors:** Xinyu Yang, Ke Lin, Shanmin Ni, Jianmin Wang, Qingqing Tian, Huaijun Chen, Matthew A. Brown, Kaidi Zheng, Weitao Zhai, Li Sun, Shengwei Jin, Jianguang Wang

**Affiliations:** 1 Department of Medicinal Chemistry, School of Pharmaceutical Sciences, Wenzhou Medical University, Wenzhou, China; 2 Department of Biochemistry, School of Basic Medical Sciences, Wenzhou Medical University, Wenzhou, 325035 China; 3 Department of Rheumatology, Jiamusi Central Hospital, Jiamusi, China; 4 Institute of Health and Biomedical Innovation, Translational Research Institute, Queensland University of Technology, Princess Alexandra Hospital, Brisbane, Australia; 5 Centre for Precision Medicine, The First Affiliated Hospital of Wenzhou Medical University, Wenzhou, China; 6 Department of Orthopaedic Surgery, Shanghai Guanghua Special Hospital for Rheumatoid Arthritis, Shanghai, China; 7 Department of Immunology and Rheumatology, The First Affiliated Hospital of Wenzhou Medical University, Wenzhou, 325035 China; 8 Department of Anesthesia and Critical Care, The Second Affiliated Hospital of Wenzhou Medical University, Wenzhou, 325035 China

**Keywords:** Rheumatoid arthritis, CTGF, ACPA, Rheumatoid factor, Biomarker

## Abstract

**Background:**

Our previous proteomic study indicated that connective tissue growth factor (CTGF) may be a potential biomarker for rheumatoid arthritis (RA) diagnosis. The aim was to assess the performance of CTGF as a biomarker of RA.

**Method:**

Serum and synovial fluid CTGF was detected using a direct high sensitivity sandwich ELISA kit. Serum CTGF levels were tested for discriminatory capacity and optimal assay cutoffs determined in a training cohort of 98 cases of RA with 103 healthy controls. The assay performance was then validated in a further cohort of 572 patients (with RA (*n* = 217), ankylosing spondylitis (*n* = 92), gout (*n* = 74), osteoarthritis (*n* = 52), systemic lupus erythematosus (*n* = 72), or primary Sjögren’s syndrome (pSS) (*n* = 65)).

**Results:**

Significant elevation of synovial fluid CTGF concentration was found in RA patients, demonstrating excellent diagnostic ability to predict RA (area under the curve (AUC) = 0.97). Similar results were found in serum CTGF detection. At the optimal cutoff value 88.66 pg/mL, the sensitivity, specificity, and the AUC was 0.86, 0.92, and 0.92, respectively, in the training cohort. Similar performance was observed in the validation cohort, with sensitivity, specificity, positive likelihood, and negative likelihood of 0.82, 0.91, 5.74, and 0.12, respectively. Stronger discriminatory capacity was seen with the combination of CTGF and anti-citrullinated protein antibody (ACPA) (AUC = 0.96) than with either ACPA or rheumatoid factor (RF) alone (AUC = 0.80 or 0.79, respectively). The discriminatory performance of serum CTGF was consistent across all inflammatory conditions tested (AUC >0.92 in all cases), with the sole exception of pSS. Serum CTGF did not vary with symptom duration or disease activity.

**Conclusions:**

Serum CTGF is a promising diagnostic biomarker for RA, with performance in the current study better than either ACPA or RF.

**Electronic supplementary material:**

The online version of this article (doi:10.1186/s13075-017-1463-1) contains supplementary material, which is available to authorized users.

## Background

Rheumatoid arthritis (RA), which is the most common chronic inflammatory joint disease that affects approximately 1% of the world’s population [1], is characterized by synovial joint inflammation, progressive joint destruction, and disability [2]. Currently, the clinical diagnosis of RA mainly relies on joint involvement, acute-phase reactants, duration of symptoms, and serological indices, including traditional rheumatoid factor (RF) and the presence of anti-citrullinated protein antibody (ACPA) [3].

However, in clinical use the 2010 criteria remain deficient, especially the serological indices. First, serological indices lack sensitivity or specificity. According to the meta-analysis of Nishimura et al., the sensitivity and specificity of a new serum index ACPA for the diagnosis of RA were 67% and 95%, respectively, and the sensitivity and specificity of the traditional index RF were even lower at 69% and 85% [4], respectively. As ACPA has high specificity, and RF has relatively higher sensitivity, the current recommendation is to combine RF and ACPA to detect RA. This combination truly improves the diagnostic value of these tests. However, studies have found that even the combination of these two markers is not perfect, with sensitivity of 78% and specificity of 82% [5] to detect RF-positive or ACPA-positive patients.

The second problem is the poor detection of early RA (ERA) by ACPA, with sensitivity as low as 57%. Similarly, the combination of ACPA and RF has limited performance benefits over either individual index [6, 7]. Therefore, the search for new serum biomarkers, especially those with high specificity and sensitivity to improve the current diagnostic tests for RA, retains great significance.

Connective tissue growth factor (CTGF) was first discovered by Bradham in 1991 [8] and belongs to the CCN family of growth factors, named CCN2. It is a 38 kD cysteine-rich protein made up of four domains, including insulin-like growth factor binding protein (IGFBP), von Willebrand factor type C repeat (VWC), thrombospondin type 1 repeat (TSP1), and C-terminal cystine-knot (CT) modules [9]. CTGF plays an important role in many physiological and pathological activities [10], such as inflammation, angiogenesis, wound healing, fibrosis, carcinogenesis, and tumor development [11]. There is some evidence that CTGF could also be involved in the onset of RA. Nozawa identified increased expression of CTGF in serum from 39 patients with RA when compared to patients with osteoarthritis (OA) and further confirmed that CTGF could enhance the activity of osteoclasts by stimulating integrin protein α_v_β_3_ to aggravate bone destruction [12]. Moreover, Fujishiro et al. found that inhibiting CTGF by neutralizing the anti-CTGF monoclonal antibody (mAb) significantly ameliorated arthritis in a murine model of RA [13]. In addition, our previous proteomic study [14] and the subsequent validation tests using PCR and western blot found that CTGF was significantly elevated in the synovial fibroblasts of 50 patients with RA in comparison with 50 healthy controls, raising the possibility that it could be a potential diagnostic biomarker for RA. To evaluate the diagnostic value of CTGF, we performed a multicenter validation cohort study to determine the discriminatory value of CTGF in RA diagnosis.

## Methods

### Patients and samples

Cases and healthy controls were recruited from the First Affiliated Hospital of Wenzhou Medical University, the Central Hospital of Jiamusi City, and Shanghai Guanghua Hospital from 7 September 2010 to 31 September 2016. The sources and the numbers of samples are shown in Additional file 1. RA diagnosis was defined according to the 2010 American College of Rheumatology (ACR) criteria. A “training set” consisting of 98 patients with RA and synovial fluids from 70 patients with RA were collected. A validation cohort of 572 patients was also used; this included 217 patients (38%) diagnosed with RA (according to the 2010 ACR criteria for RA), 92 (16%) with ankylosing spondylitis (AS) (Modified New York criteria for AS), 74 (13%) with gouty arthritis (gout) (2015 ACR criteria for gout), 52 (9%) with OA (1986 ACR criteria for OA of the knee), 72 (13%) with systemic lupus erythematosus (SLE) (1997 ACR criteria for SLE), and 65 (11%) with primary Sjögren’s syndrome (pSS) (2012 ACR criteria for pSS). Detailed demographic and clinical characteristics of the different groups are shown in Table 1 and Additional file 2.

**Table 1:** Demographic and clinical characteristics of participants in two cohorts

	Training cohort		Validation cohort	
	RA (before 1 May 2016)	Control (before 1 May 2016)	RA (after 16 May 2016)	non-RA (after 16 May 2016)
Sex (female/male), *n*	80/18	50/53	174/43	187/168
Age (years)	58 (27, 85)	49 (19, 86)	59 (15, 84)	48 (13, 94)
Symptom duration (years)	10 (0.01, 50)	NA	5 (0.01, 50)	4 (0.01, 50)
CRP (mg/L)	22 (0.6, 331)	NA	18.6 (0.16, 339)	8.0 (0.16, 436)
ESR (mm/h)	44 (2, 140)	NA	42 (2, 134)	24 (2, 120)
ACPA positive, *n* (%)	71 (72)	NA	147(68)	12 (3)
RF positive, *n* (%)	65 (66)	NA	136 (63)	63 (18)
Serum CTGF (pg/mL)	293.9 (7.87, 1285)	30 (0.16, 171.3)	289.2 (1.82, 2351)	22.45 (0.12, 541)
Synovial fluid CTGF (pg/mL)	534 (1.55, 2574)	23.87 (2.65, 166.8)	NA	NA

Values are expressed as median (minimum, maximum) unless state otherwise

*Abbreviations*: *RA* rheumatoid arthritis, *NA* not applicable, *CRP* C-reactive protein, *ESR* erythrocyte sedimentation rate, *ACPA* antibodies directed against citrullinated peptides, *RF* rheumatoid factor, *CTGF* connective tissue growth factor

All patients were included in the study on the first day of clinical admission; serum was collected from the patients before they underwent any treatment. Synovial fluid was collected during joint arthroplasty surgery from both patients with RA and patients with femoral neck fracture or meniscus injury (control subjects) from Shanghai Guanghua Hospital. The Clinical Research Ethics Committees of the First Affiliated Hospital of Wenzhou Medical University (No. 2016157), the Central Hospital of Jiamusi City (No. 2012010), and Shanghai Guanghua Hospital (No. 200903) approved the study. All of the subjects provided written informed consent.

### CTGF concentration detected by ELISA

CTGF concentrations were detected by direct high-sensitivity sandwich ELISA (Human CTGF ELISA Kit, GWB-SKR010, GenWay Biotech Inc., USA) in accordance with the manufacturer’s instructions. Absorption was determined at an optical density of 450 nm. The data were analyzed directly. ACPA and RF were measured in the clinical laboratory. All of the reactions were conducted in triplicate. Detailed kit information and specifications are provided in Additional file 3.

### Statistical analysis

All data were calculated for quantitative variables in SPSS (version 19.0, IBM, USA). The Shapiro-Wilk method was used to test whether the data were normally distributed, and the Levene method was used to test the homogeneity of variance. Two sets of data that did not meet the criteria for normal distribution and homogeneity of variance were analyzed by the Mann-Whitney *U* test.

The discriminatory ability of CTGF was assessed by plotting the receiver operating characteristic (ROC) curve based on data from the training cohort. Detailed diagnostic performance of CTGF to identify RA was evaluated according to the area under the curve (AUC), sensitivity, and specificity. Confidence intervals (95%) for the AUC were performed in GraphPad Prism (version 5, GraphPad Software, USA), and the cutoff point of serum CTGF for predicting RA was chosen using Youden’s index.

## Results

### Diagnostic performance of CTGF for detection of RA

**Fig. 1:**
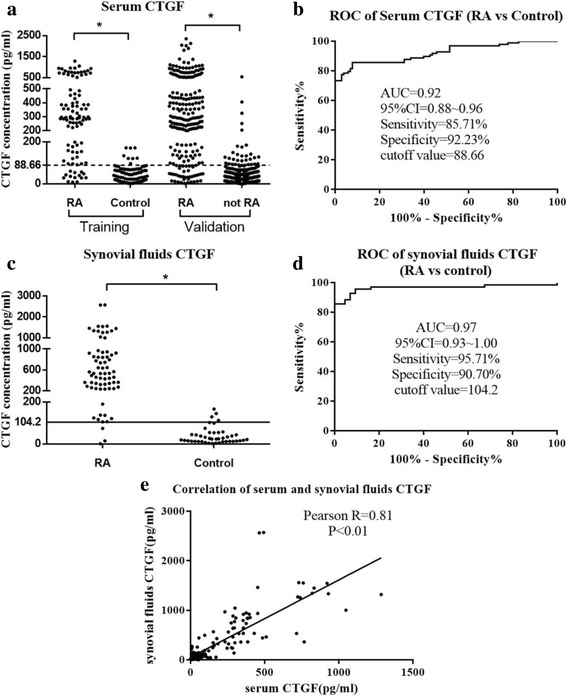
Diagnostic performance of serum connective tissue growth factor (CTGF) and synovial fluid CTGF. **a** Serum CTGF in the training cohort. CTGF concentration was detected in serum samples from patients with rheumatoid arthritis (RA) (*n* = 98) and the control group (*n* = 103) using a CTGF ELISA kit. The black horizontal dotted line represents the cutoff value of 88.66 pg/mL and the asterisks represent statistical differences (*p* < 0.01). **b** Receiver operating characteristic (ROC) curve analysis of serum CTGF for diagnosis of RA. At the cutoff 88.66 pg/mL, the sensitivity and specificity of CTGF are 0.86 and 0.92, respectively. The AUC for CTGF is provided with its associated 95% confidence intervals. **c** Synovial fluid CTGF in the RA and control groups. Synovial fluid CTGF was detected using the CTGF ELISA kit, and the absorption rate was determined at an optical density of 450 nm. The black horizontal solid line represents the cutoff value of control at 104.2 pg/mL; asterisks represent statistical differences (*p* < 0.01). **d** ROC curve analysis of serum CTGF for diagnosis of RA. **e** Correlation between serum CTGF and synovial fluid CTGF. Linear correlation was analyzed, and a strong association was observed, with Pearson *R* of 0.81 (*p* < 0.01)

In serum, CTGF concentrations in patients with RA were significantly higher compared to the controls (*p* < 0.05). (Fig. 1a and Table 1) Serum CTGF had high diagnostic value for RA in terms of sensitivity, specificity, and the AUC, which were 0.86, 0.92, and 0.92, respectively, at the cutoff value of 88.66 pg/mL (Youden’s index, 0.78; Fig. 1b).

RA control subjects were mostly recruited at the First Affiliated Hospital of Wenzhou Medical University and Shanghai Guanghua Hospital. There were no significant differences in the CTGF concentrations in subject samples from the two centers (*p* > 0.05). ROC analysis showed similar predictive ability in subject samples from the two centers (Additional file 4).

As synovial fluid examinations could be supplements for serological tests, we measured CTGF in synovial fluid from patients with RA and control subjects. Compared to the healthy controls (23.87 (2.65, 166.8) pg/mL), patients with RA (534 (1.55, 2574) pg/mL) had a higher concentration of synovial fluid CTGF (Fig. 1c and Table 1). The diagnostic value of synovial fluid CTGF for RA was even higher (cutoff value, 104.2 pg/mL; Youden's index, 0.86), as the sensitivity, specificity, and AUC were 0.96, 0.91, and 0.97, respectively (Fig. 1d).

As both serum CTGF and synovial fluid CTGF could be good diagnostic indicators, the correlation between them was then calculated. Strong association was observed between serum CTGF and synovial fluid CTGF, with *R* = 0.81 (*p* < £0.01; Fig. 1e). This suggests that they are from the same source.

### Validation of the high diagnostic value of CTGF in the validation cohort

We then sought to validate the serum CTGF threshold 88.66 pg/mL as a biomarker for RA, and test its discriminatory performance to distinguish between RA and various inflammatory conditions. Of the 572 patients in the validation study, 209 were CTGF-positive, and 178 of these had RA according to the ACR criteria. There were 39 patients with RA who were missed by the CTGF test. The positive and negative predictive values were 0.85 and 0.90, respectively. Other evaluation parameters demonstrated similarly high diagnostic value for CTGF, with sensitivity, specificity, positive likelihood ratio (+LR), and negative likelihood ratio (−LR) of 0.82, 0.91, 5.74, and 0.12, respectively (Table 2).

**Table 2:** Evaluation of the diagnostic experiment using CTGF in the validation cohort

Tests^a^	Sensitivity	Specificity	Positive likelihood ratio	Negative likelihood ratio	Positive predictive value	Negative predictive value	Youden index
CTGF test in all participants^b^	0.82	0.91	5.74	0.12	0.85	0.90	0.73
CTGF test in ACPA-negative participants^c^	0.76	0.91	1.71	0.05	0.63	0.95	0.67
CTGF test in RF-negative participants^d^	0.79	0.91	2.46	0.06	0.71	0.94	0.70

*CTGF* connective tissue growth factor, *ACPA* anti-citrullinated protein antibodies, *RF* rheumatoid factor

^a^Diagnostic experiments using CTGF with the cutoff 88.66 pg/mL, which was obtained from the training cohort data as described previously

^b^Evaluation of the CTGF test in all participants in the validation cohort

^c^Evaluation of the CTGF test in participants in the validation cohort who were ACPA-negative

^d^Evaluation of the CTGF test in participants in the validation cohort who were RF-negative

In the same validation cohort, ACPA had sensitivity and specificity of 67% and 97%, respectively. When used at its clinically utilized cutoff (>50 U/mL), a total of 82 patients were incorrectly classified. We then calculated the supplementary function of CTGF in detecting RA in ACPA-negative participants. The sensitivity, specificity, +LR, and –LR were 0.76, 0.91, 1.71, and 0.05, respectively. Similarly, the sensitivity, specificity, +LR, and –LR of CTGF in detecting RA from RF-negative participants were 0.79, 0.91, 2.46, and 0.06, respectively (Table 2).

### Important differential diagnostic ability of CTGF

The performance of serum CTGF was tested to discriminate between patients with RA and the diagnostic subsets in the validation cohort. Based on the ROC analysis comparing patients with RA to patients in each subset of the non-RA group (Table 3), the optimum diagnostic cutoff was 73.35 pg/mL (Youden’s index, 0.80) for AS; 84.42 pg/mL (Youden’s index, 0.82) for gout; 46.75 pg/mL (Youden’s index, 0.79) for OA; 151 pg/mL (Youden’s index, 0.66) for PSS; and 79.64 pg/mL (Youden’s index, 0.82) for SLE. These results demonstrate the significant differential diagnostic ability of CTGF in distinguishing these diseases.

**Table 3:** Differential diagnosis of RA and other rheumatic diseases using cutoff points for serum CTGF and ACPA

		AUC (95% CI)	S.e.	Cutoff	Sensitivity %	Specificity %	*P* value
RA vs AS	CTGF	0.94 (0.91, 0.97)	0.01	73.35	86.18	93.48	<0.0001
	ACPA	0.88 (0.84, 0.92)	0.01	34.96	77.42	90.22	<0.0001
RA vs gout	CTGF	0.95 (0.93, 0.98)	0.01	84.42	84.33	97.3	<0.0001
	ACPA	0.89 (0.86, 0.93)	0.02	49.23	68.2	98.65	<0.0001
RA vs OA	CTGF	0.94 (0.92, 0.97)	0.01	46.75	88.48	90.38	<0.0001
	ACPA	0.89 (0.85, 0.93)	0.02	42.33	71.89	94.23	<0.0001
RA vs PSS	CTGF	0.86 (0.82, 0.90)	0.02	151	75.12	90.77	<0.0001
	ACPA	0.83 (0.78, 0.88)	0.03	43.46	71.43	90.77	<0.0001
RA vs SLE	CTGF	0.92 (0.89, 0.95)	0.02	79.64	85.71	95.83	<0.0001
	ACPA	0.84 (0.79, 0.88)	0.02	76.56	65.44	98.61	<0.0001

*P* value compared with Null hypothesis: true area % 0.5

*Abbreviations*: *AUC* area under the receiver operating characteristic curve, *95% CI* 95% confidence interval, *S.e.* standard error, *RA* rheumatoid arthritis, *AS* ankylosing spondylitis, *OA* osteoarthritis, *pSS* primary Sjögren’s syndrome, *SLE* systemic lupus erythematosus, *ACPA* anti-citrullianted protein antibodies, *CTGF* connective tissue growth factor

### No significant correlation between serum CTGF and RA symptom duration or activity

**Fig. 2:**
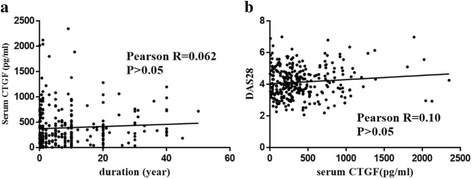
Serum concentration of connective tissue growth factor (CTGF) is not associated with rheumatoid arthritis (RA) symptom duration or the disease activity score in 28 joints (DAS28). **a** No significant association between symptom duration and serum CTGF concentration. All patients with RA (including patients with RA in the training and validation cohorts) were pooled in the final analysis, and the correlation between serum CTGF and symptom duration was tested. No significant correlation was observed, with *R* = 0.062 (*p* > 0.05). **b** No significant correlation between DAS28 and serum CTGF concentration. All patients with RA were pooled in the final analysis, and the correlation between serum CTGF and DAS28 was tested. No significant correlation was observed, with *R* = 0.10 (*p* > 0.05)

Again in the pooled cases of RA from the training and validation cohorts, the correlation between serum CTGF and symptom duration or the disease activity score in 28 joints (DAS28) was calculated. No significance was observed, with *R* = 0.062, *p* > 0.05 and *R* = 0.10, *p* > 0.05, respectively (Fig. 2).

No association between serum CTGF and gender was observed in patients with RA (*p* > 0.05).

### Addition of serum CTGF assay improves diagnostic performance of ACPA and RF

**Fig. 3:**
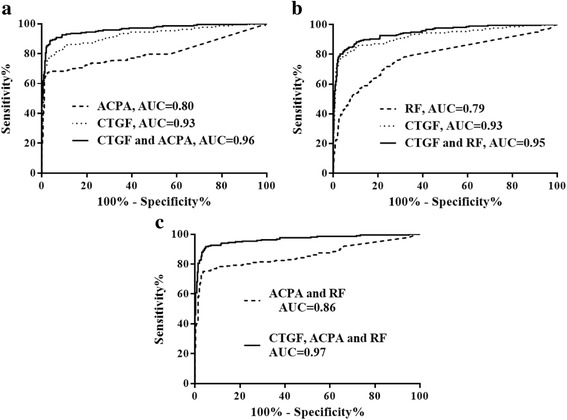
Serum indicators and combinations of serum indicators for diagnosis of rheumatoid arthritis (RA). **a** Receiver operating characteristic (ROC) analysis of connective tissue growth factor (CTGF), anti-citrullinated protein antibodies (ACPA), and their combination for diagnosing RA. Serum ACPA concentrations were tested in all participants recruited to the validation cohort. ROC analysis was carried out, and the area under the curve (AUC) for CTGF and ACPA was 0.93 and 0.80, respectively. The combination of CTGF and ACPA further improved the diagnostic ability for RA with an AUC of 0.96. **b** ROC analysis for CTGF, RF, and their combination for diagnosing RA. Serum rheumatoid factor (RF) concentrations were tested for all participants recruited to the training cohort. ROC analysis was carried out, and the AUC for CTGF and RF, and for their combination, was 0.93, 0.8, and 0.95, respectively. **c**. ROC analysis of the combinations of ACPA and RF, and CTGF, ACPA, and RF for diagnosing RA. We tested the diagnostic value of the currently recommended serum assay, and the AUC for the combination of ACPA and RF was 0.86, while the AUC for adding CTGF to the combined ACPA and RF was significantly increased to 0.97

ROC analysis on a combination of serum indicators was then processed in the patients with RA from the validation cohorts (Fig. 3). While the AUC of single indicators of CTGF, ACPA, and RF was 0.93, 0.80, and 0.79 respectively, the AUC of the combination of CTGF and ACPA, CTGF and RF, ACPA and RF, and CTGF, ACPA, and RF were 0.96, 0.95, 0.86, and 0.97, respectively. The numbers of ACPA/CTGF-positive, RF/CTGF-positive, RF/ACPA-positive, triple-positive, and single-positive subjects vs all negative subjects are shown in Additional file 5.

## Discussion

RA is a chronic autoimmune disease with symmetric polyarthritis as one of its main manifestations. Diagnosis can be challenging, particularly in early disease and in patients with atypical presentation [15]. Thus, much research and effort have gone into the development of RA diagnosis criteria in order to improve the diagnosis and classification of RA. The 1987 ACR criteria for RA, comprising morning stiffness, arthritis, rheumatoid nodules, serum RF, and radiographic changes, were in use for many years [16]. However, these criteria are not very efficient, in that the criteria only help to diagnose those patients who already have serious structural damage. In addition, some of the criteria, like radiographic changes, are not accurate quantitative indicators and mainly depend on the subjective judgment of rheumatologists [17]. These weaknesses stimulated the development of the current gold standard, the 2010 ACR criteria for RA. Compared to the previous version, the new criteria did not include the subjective and inaccurate criterion of radiographic changes, but included a more specific and objective biomarker, ACPA. A systematic literature review shows that the 2010 criteria help to diagnose more patients in the earlier stages of RA. The sensitivity of these criteria has risen by 11%, at the cost of a decline in specificity of only 4% [18].

There remains a need for improved diagnostic tests and criteria in RA, in particular for identifying the disease and to assist in distinguishing RA from other rheumatic diseases. In the current study, we demonstrated that CTGF could partially solve this problem.

One of the most important problems in RA diagnosis is a lack of sensitivity or specificity of the current indicators, ACPA and RF. ACPA is a good clinical indicator of RA (high predictability values and high diagnostic accuracy) and has moderate sensitivity of approximately 67% [4, 19]. However, the sensitivity of ACPA is lower in early RA. RF fares even worse, with a range of specificity from 38% to 85%, indicating that positive IgM-RF has modest diagnostic value [4, 20]. In the current study, we demonstrated that serum CTGF was an excellent serum indicator, with better performance than either of these widely used assays. As an independent biomarker, the sensitivity, specificity, and AUC of CTGF appeared high at 0.86, 0.92, and 0.92, which was much better than for RF, and CTGF had similar specificity but better sensitivity when compared with ACPA. The fact that ACPA is part of the ACR 2010 classification criteria would be expected to inflate its performance to diagnose RA using these criteria, and the relative superiority of serum CTGF in the current study is notable. Furthermore, approximately 30% of patients with RA who have clinical manifestations of the disease may have negative ACPA results [21]. Therefore, we tested the diagnostic ability of CTGF in the ACPA-negative population, and found that serum CTGF was able to identify more patients with RA, with sensitivity and specificity of 76% and 91%, respectively.

The combination of RF and ACPA performs significantly better than either marker alone in the diagnosis of RA [5, 22]. However, researchers also reported that a model including ACPA and RF can correctly identify only 54–57% of patients with RA [22]. In our study, a combination of CTGF, ACPA, and RF had the best diagnostic efficiency, better than single indicators, the combination of RF and CTGF, or even the recommended assay of combined ACPA and RF, with an AUC of 0.97. However, as the AUC of the combination of CTGF and ACPA was not significantly weaker than the combination of the three, we recommend the use of the combination of CTGF and ACPA.

Biomarkers are also of clinical utility in distinguishing RA from other rheumatic diseases. Matsui et al. [23] reported a relatively high frequency of ACPA in patients with other rheumatic diseases, including SLE (15%), pSS (14%), polymyositis/dermatomyositis (23%), and scleroderma (16%). In the present study, we found serum CTGF has good discriminatory capacity in distinguishing RA from other rheumatic diseases, with the AUC for serum CTGF detection greater than 0.92 for all diseases tested except for pSS. The discriminatory capacity of serum CTGF is extremely high in distinguishing SLE from RA and OA from RA.

Synovial proliferation and joint erosion are characteristic features of RA [24, 25]. CTGF has been shown to stimulate hyperproliferation of fibroblast-like synoviocytes in RA [26], and to act as an angiogenesis factor in the formation of pannus. These roles may explain why CTGF discriminates well between RA and other inflammatory arthropathies, and demonstrates face validity for the biomarker.

There were some negative results in our study that might contribute to the application of CTGF in diagnosing RA. No correlation was observed between serum concentrations of CTGF and the duration of RA symptoms. This indicated that the diagnostic efficiency of CTGF may not decrease in early RA, whereas there could be a significant decline in the sensitivity of ACPA when diagnosing patients with ERA rather than patients with established RA [7]. Interestingly, almost no association of CTGF with DAS28 was found in our study, indicating that CTGF might be used without too much consideration of different disease activities.

Synovial fluid detection could also give some clues in distinguishing RA from diseases that are difficult to identify. We quantified CTGF in synovial fluid from patients with RA and the control subjects, and we found that this was a better biomarker than CTGF in serum, with a higher AUC for diagnosing RA. Additionally, the RA-to-control ratio of the concentration of CTGF in synovial fluid and serum are 60 and 10, respectively, and in synovial tissue, according to our previous proteomic study the ratio was 2.54 [14]. Furthermore, there is strong correlation between serum CTGF and synovial fluid CTGF, indicating the same sources of CTGF production. Thus, we surmised that rather than the chondrocytes [27], the synovial tissue or the synovial fibroblasts [12] in the tissue was the initial source of CTGF, and CTGF was then secreted into the synovial fluid, participating in pannus formation as an angiogenesis factor [28], and later diffused into the blood. This hypothesis needs further validation.

Our study has several limitations. Although we have carried out a multicenter study, our participating centers were all from eastern or northern areas of China, and therefore further studies in different ethnic groups are warranted. We also have not tested the performance of CTGF in a specific cohort of patients with early arthritis, nor in preclinical disease, which are situations where ACPA has demonstrated diagnostic or predictive utility. Further testing of the impact of treatment on CTGF levels is also indicated.

## Conclusions

In summary, in a multicenter study we identified an excellent and stable biomarker for RA. Serum CTGF concentration has much better diagnostic ability than RF, and is not weaker than ACPA. The combination of CTGF and ACPA could further improve diagnostic efficiency. In addition, it is a better biomarker to distinguish RA from other rheumatic diseases. Thus, we recommend CTGF for clinical use in diagnosing RA and distinguishing RA from other rheumatic diseases.

## Additional files

Additional file 1: Table S1. Sample numbers and sources in the training and validation cohort. (DOCX 14 kb)

Additional file 2: Table S2. Detailed demographic and clinical characteristics of patients in the validation cohort with conditions other than RA (not-RA). (DOCX 15 kb)

Additional file 3: Table S3. Detailed kit information for assays of CTGF, ACPA, and RF. (DOCX 14 kb)

Additional file 4: Figure S1. ROC analysis showed the similar predictive performance at the two centers. (DOCX 230 kb)

Additional file 5: Table S4. The diagnosis value of ACPA, RF, and CTGF in the validation cohort. (DOCX 13 kb)

## Abbreviations

ACPA: Anti-citrullinated protein antibody
ACR: American College of Rheumatology
AS: Ankylosing spondylitis
AUC: Area under the curve
CT: C-terminal cystine-knot
CTGF: Connective tissue growth factor
DAS28: Disease activity score in 28 joints
ELISA: Enzyme-linked immunosorbent assay
ERA: Early rheumatoid arthritis
Gout: Gouty arthritis
IGFBP: Insulin-like growth factor binding protein
LR: Likelihood ratio
OA: Osteoarthritis
pSS: Primary Sjögren’s syndrome
RA: Rheumatoid arthritis
RF: Rheumatoid factor
ROC: Receiver operating characteristic
SLE: Systemic lupus erythematosus
TSP1: Thrombospondin type 1 repeat
VWC: von Willebrand factor type C repeat
