# Supplementary material for: Serum connective tissue growth factor is a highly discriminatory biomarker for the diagnosis of rheumatoid arthritis
Source: Arthritis Res Ther. 2017 Nov 22;19:257. doi: 10.1186/s13075-017-1463-1 (PMC5700625; doi:10.1186/s13075-017-1463-1)
Supplement: Supplementary file 1 — Sample numbers and sources in the training and validation cohort. (DOCX 14 kb) [file 13075_2017_1463_MOESM1_ESM.docx]

Additional file1: Table S1. Sample numbers and sources in training and validation cohort.

| Cohorts | Diseases | Serum samples | | |  |  | Synovial fluids |
| --- | --- | --- | --- | --- | --- | --- | --- |
|  |  | The first affiliated hospital of Wenzhou Medical University | The central hospital of Jiamusi City | Shanghai Guanghua hospital | Total patients |  | Shanghai Guanghua hospital |
| Training cohort | Normal | 39 | 21 | 43 | 103 |  | 43 |
|  | RA | 28 | 0 | 70 | 98 |  | 70 |
| Validation cohort | RA | 176 | 41 | 0 | 217 |  | 0 |
|  | AS | 91 | 1 | 0 | 92 |  | 0 |
|  | Gout | 53 | 21 | 0 | 74 |  | 0 |
|  | OA | 30 | 22 | 0 | 52 |  | 0 |
|  | PSS | 53 | 12 | 0 | 65 |  | 0 |
|  | SLE | 50 | 22 | 0 | 72 |  | 0 |

Abbreviations: RA=Rheumatoid arthritis, AS=Ankylosing spondylitis, Gout=Gouty arthritis, OA=Osteoarthritis, PSS=Sjogren's syndrome, SLE=Systemic lupus erythematosus.
