# Supplementary material for: Serum connective tissue growth factor is a highly discriminatory biomarker for the diagnosis of rheumatoid arthritis
Source: Arthritis Res Ther. 2017 Nov 22;19:257. doi: 10.1186/s13075-017-1463-1 (PMC5700625; doi:10.1186/s13075-017-1463-1)
Supplement: Supplementary file 2 — Detailed demographic and clinical characteristics of patients in the validation cohort with conditions other than RA (not-RA). (DOCX 15 kb) [file 13075_2017_1463_MOESM2_ESM.docx]

Additional file2: Table S2. Detailed demographic and clinical characteristics of not-RA in the validation cohort

|  | AS | GOUT | OA | PSS | SLE |
| --- | --- | --- | --- | --- | --- |
| Sex(F/M) | 21/71 | 7/67 | 33/19 | 62/3 | 64/8 |
| Age(Years) | 40.5[13,74] | 59.5[14,94] | 62[23,87] | 52[15,78] | 40[13,72] |
| Symptom duration | 3.5[0.01, 31] | 8.5[0.01,50] | 3[0.01,20] | 3[0.02,20] | 5[0.04,30] |
| CRP(mg/L) | 14.05[0.53, 289] | 21.7[0.9,436] | 4.02[0.17,118] | 1.81[0.16,59.9] | 8.53[0.16,181] |
| ESR(mm/h) | 29[2,93] | 29[3,88] | 18[2,89] | 23[2,120] | 21[2,69] |
| ACPA positive(%) | 1(1) | 1(1) | 0(0) | 6(10) | 4(6) |
| RF positive(%) | 27(29) | 12(16) | 5(1) | 13(20) | 6(8) |
| HLA-B27 positive(%) | 83(90) | NA | NA | NA | NA |
| Serum CTGF(pg/mL) | 14.7[0.12, 541.0] | 12.26[0.48, 109.5] | 20.46[0.84, 124.6] | 57.84[1.36, 404.9] | 31.86[0.30, 116.8] |

Values are expressed as Median[Minimum, Maximum].

Abbreviations: CRP=C reactive protein; ESR=Erythrocyte sedimentation rate; ACPA=Antibodies directed against citrullinated peptides; RF=Rheumatoid factor.
