# Supplementary material for: Serum connective tissue growth factor is a highly discriminatory biomarker for the diagnosis of rheumatoid arthritis
Source: Arthritis Res Ther. 2017 Nov 22;19:257. doi: 10.1186/s13075-017-1463-1 (PMC5700625; doi:10.1186/s13075-017-1463-1)
Supplement: Supplementary file 3 — Detailed kit information for assays of CTGF, ACPA, and RF. (DOCX 14 kb) [file 13075_2017_1463_MOESM3_ESM.docx]

Additional file3: Table S3. Detailed kit information of CTGF, ACPA and RF

|  | CTGF | ACPA | RF |
| --- | --- | --- | --- |
| Kit | Human CTGF ELISA Kit, GWB-SKR010, | Elecsys and cobas e analyzers, Anti-CCP Kit 05031656, | N Latex RF Kit OPCEG03E0502, BN* II/BN ProSpec® System, |
| Company | GenWay Biotech Inc., | Roche Diagnostics GmbH, | Siemens Healthcare Diagnostics Products GmbH, |
| Country | USA | Germany | Germany |
| Standard range | 62-4000pg/ml | 7-500U/ml | 10-640IU/ml |
| Application | Research | Diagnostic | Diagnostic |
