# Supplementary material for: Serum connective tissue growth factor is a highly discriminatory biomarker for the diagnosis of rheumatoid arthritis
Source: Arthritis Res Ther. 2017 Nov 22;19:257. doi: 10.1186/s13075-017-1463-1 (PMC5700625; doi:10.1186/s13075-017-1463-1)
Supplement: Supplementary file 4 — ROC analysis showed the similar predictive performance at the two centers. (DOCX 230 kb) [file 13075_2017_1463_MOESM4_ESM.docx]

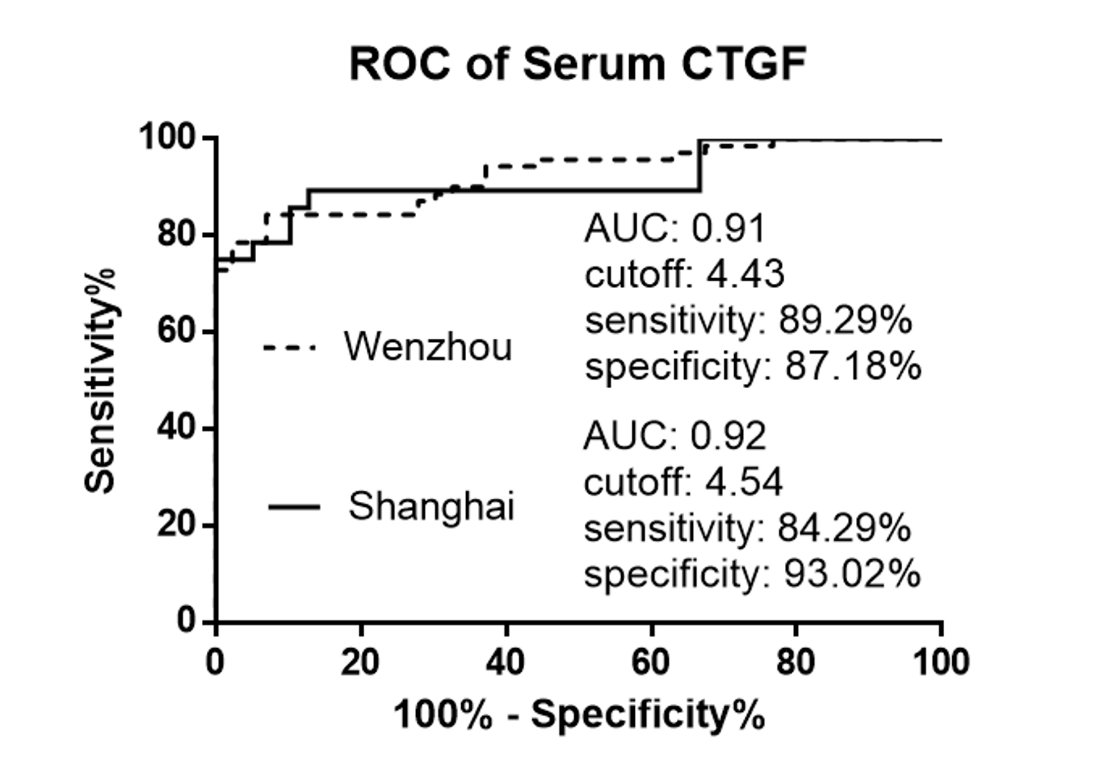


Additional file 4: Figure S1. ROC analysis showed the similar predictor performance between the two centres
