# Supplementary material for: Serum connective tissue growth factor is a highly discriminatory biomarker for the diagnosis of rheumatoid arthritis
Source: Arthritis Res Ther. 2017 Nov 22;19:257. doi: 10.1186/s13075-017-1463-1 (PMC5700625; doi:10.1186/s13075-017-1463-1)
Supplement: Supplementary file 5 — The diagnosis value of ACPA, RF, and CTGF in the validation cohort. (DOCX 13 kb) [file 13075_2017_1463_MOESM5_ESM.docx]

Additional file 5: Table S4. The diagnosis value of ACPA, RF, and CTGF in the validation cohort

|  | RA(n=217) | non-RA(n=355) |
| --- | --- | --- |
| ACPA+ | 147 | 12 |
| RF+ | 136 | 63 |
| CTGF+ | 178 | 31 |
| RF / CTGF+ | 114 | 5 |
| ACPA / CTGF+ | 125 | 2 |
| RF / ACPA+ | 107 | 3 |
| CTGF/RF / ACPA- | 12 | 259 |
| CTGF/RF / ACPA+ | 90 | 0 |
